# Status and Challenges in Homogenization Methods for Lattice Materials

**DOI:** 10.3390/ma15020605

**Published:** 2022-01-14

**Authors:** Jacobs Somnic, Bruce W. Jo

**Affiliations:** Advanced Dynamics, Mechatronics and Collaborative Robotics (ADAMS) Laboratory, Department of Mechanical Engineering, State University of New York (SUNY), Incheon 21985, Korea; jacobs.somnic@stonybrook.edu

**Keywords:** homogenization method, lattice materials, periodic cellular materials, multiscale mechanics

## Abstract

Lattice structures have shown great potential in that mechanical properties are customizable without changing the material itself. Lattice materials could be light and highly stiff as well. With this flexibility of designing structures without raw material processing, lattice structures have been widely used in various applications such as smart and functional structures in aerospace and computational mechanics. Conventional methodologies for understanding behaviors of lattice materials take numerical approaches such as FEA (finite element analysis) and high-fidelity computational tools including ANSYS and ABAQUS. However, they demand a high computational load in each geometry run. Among many other methodologies, homogenization is another numerical approach but that enables to model behaviors of bulk lattice materials by analyzing either a small portion of them using numerical regression for rapid processing. In this paper, we provide a comprehensive survey of representative homogenization methodologies and their status and challenges in lattice materials with their fundamentals.

## 1. Introduction

**Figure 1:**
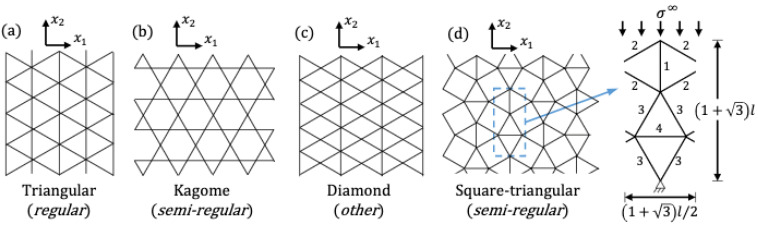
Examples of different lattice topologies: (**a**) triangular; (**b**) Kagome; (**c**) diamond; (**d**) snub square [10].

Lattice material is a cellular material consisting of a periodic network of structural elements such as rods or beams. This network of lattices exists over a wide spectrum of scale from the nanoscale to macroscale and has been applied in a wide area of applications. In the nanoscale spectrum, most of the CNT (Carbon Nano Tube) based sensors are made using lattice materials [1] as shown in Figure 1a. Micro-lattices material is being developed intensively as it offers high energy absorption capability [2,3]. On a macroscale, due to its high stiffness and lightweight properties, lattice materials are widely used in aerospace applications [4,5,6,7,8].

Lattice structures or materials could be also classified into several parameters, namely, geometry, deformation properties, and rigidity. These determine a proper approach for understanding dynamics of lattice accurately extend to design. Geometry-based classification is widely received in mathematics and solid-state physics and especially in 2-D, two main categories are considered: regular and semi-regular [9]. Representatives of each group are illustrated in Figure 1. Sub-sequentially, three types exist under the regular lattice, namely, square lattice, triangular lattice, and hexagonal lattice. In semi-regular lattices, unit cells are tessellated Later, eight semi-regular lattices are introduced in this paper for more details [9].

**Figure 2:**
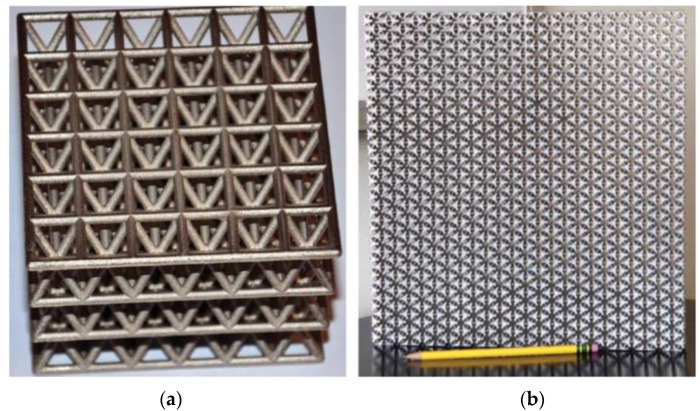
Lattice materials formed by network of beams; (**a**) ultralight Nano-metal truss hybrid lattice; (**b**) penta-mode lattice [12].

In engineering applications, spatially periodic patterns of lattices can be viewed as a material or a structure depending on its length scale. When the deformation is at a much larger length scale than the individual beam length, such a network of a lattice is defined as “lattice material”. Figure 2 shows such an example of lattice materials. On the other hand, if the length scale between deformation and the individual beam is the same, then it is viewed as a “lattice structure”. Asymptotic Theory might be a more suitable approach when we are dealing with lattice materials [11]. Meanwhile, modeling the beam individually is a better approach for lattice structure. This paper will more focus on lattice materials rather than structure as it is more relevant to the homogenization method.

**Figure 3:**
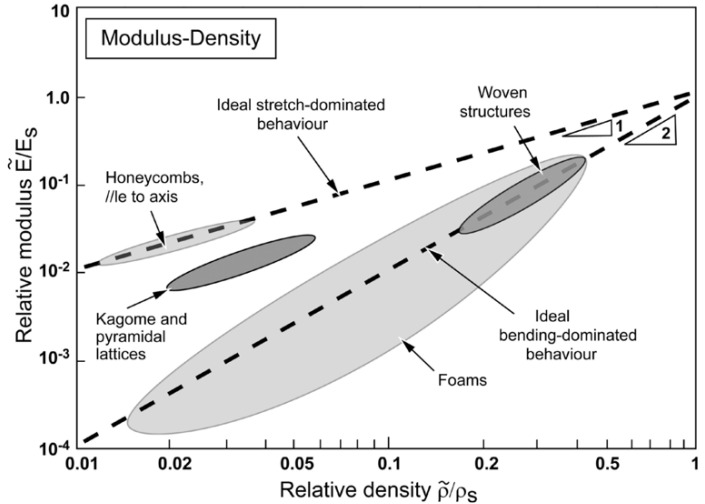
Relative modulus plotted against relative density on logarithmic scales for cellular structure [19].

The other key parameter that determines a suitable approach for understanding lattices is relative density. The relative density is defined as the density ratio of lattice material to the solid material ($\bar{\rho }=\rho^{*}/\rho_{s})$ and has a pivotal role in determining the elastostatic behavior of a lattice. Figure 3 shows the relationship between relative density and relative modulus. Slope 1 depicted in Figure 3 is for stretch-dominated lattice and slope 2 is for bending-dominated lattice. As it can be seen, honeycombs, one of the commonly used cores for sandwich panels, are extraordinarily efficient. Physically, relative density depicts the porosity of lattice material. A low value of relative density indicates high porosity, meanwhile, a high value of that indicates low porosity. For instance, $\bar{\rho }=1$ means zero porosity as the density of the lattice is the same as one of the solid or bulk. Therefore, it is crucial to employ a proper homogenization model or approach according to the value of relative density. For the low value of relative density, e.g., $\bar{\rho }<0.3$, applying Euler–Bernoulli beam or Timoshenko beam elements to model the cell-wall deformation will give an accurate result [13,14,15,16]. Furthermore, Micro-polar theory [17], Bloch Wave Analysis and Cauchy–Born hypothesis [18] might be employed for such cases as well. For a high value of relative density, the Asymptotic Homogenization method will give a better and more accurate result [11].

**Figure 4:**
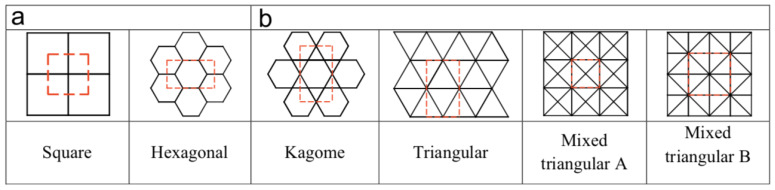
(**a**) Bending dominated lattices (**b**) Stretching dominated lattices [11].

Lattices can also be categorized into stretching-dominated or bending-dominated based on their rigidity [19]. Some representatives of both categories are shown in Figure 4. A bending-dominated lattice reacts to external loads by cell-wall bending due to its low nodal connectivity at the cell vertices. This results in a microscopic bending-dominated failure mode, where the cell elements collapse by bending stresses [18]. On the other hand, stretching-dominated lattices predominantly behave by stretching due to the high value of nodal connectivity at the cell vertices. For the same porosity or relative density, stretching-dominated lattices are stronger and have higher stiffness than bending-dominated lattices. Gibson and Ashby [20] performed structural analysis and found that the stiffness and the strength of lattice materials scale up with the value of relative density. The strength and stiffness of stretching-dominated lattice scale up linearly by its relative density $\left(\bar{\rho }\right)$, whereas the strength and stiffness of bending-dominated lattice are scaled up, respectively, by ${\bar{\rho }}^{2}$ and ${\bar{\rho }}^{1.5}$. For example, at $\bar{\rho }=0.01$, the stretching-dominated lattice is far more superior than the bending-dominated lattice as it is 100 times stiffer and 10 times stronger.

## 2. Background

For a periodic network of lattices to be considered as material, the characteristic length of its cells needs to be at least one or two orders of magnitude below the medium’s overall length scale. Hence, microscale study is vital to understand the full behavior of the structure at the global scale, which is the basic principle of the homogenization method. Numerous analytical and numerical methods have been constructed to determine the mechanical behavior of cellular materials [11,13,14,17,18,21,22,23,24,25]. All of these methods are based on various fields of physics and mathematics ranging from asymptotic theory [11], elasticity theory [13] to micro-polar theory [17]. Moreover, experimental work has been done as well [13,26,27] though it is limited in design complexity due to manufacturability in the process. However, recent advances in 3D manufacturing techniques such as 3D printing has significantly improved the production of lattice materials in terms of accuracy with various kind of solid materials. Nowadays the manufacturing process of lattice structure can be conducted at a very fine scale and with lower overall cost [28,29,30,31]. This advancement allows lattice materials to be more experimented on and be tested against existing numerical and analytical models [26,32].

The analytical works to analyze and develop a method to obtain mechanical behaviors and properties of cellular materials have been pioneered by several people; Gibson et al. [13], Masters et al. [16], Wang et al. [14], and Christensen [33]. They derived an analytical closed-form formula of mechanical properties of lattice materials for several shapes and geometry. Their method is based on one common ground assumption, which is that the cell behaves as Euler–Bernoulli beams. They obtain the mechanical properties by solving deformation and equilibrium problems for a single cell, which generates some limitations to the application of the analytical method. It could only be applied to a cell with a simple topology with small strains and no extreme change in geometry. Furthermore, it only works in lattice structures with small relative density value ($\bar{\rho }=0.3$).

In terms of computational works, several different approaches have been developed. Asymptotic Homogenization (AH) has been extensively employed to obtain the mechanical properties of lattice materials [11,34,35]. AH has been proven and validated to be an effective homogenization method through comparisons with other methods and experimental verification [8]. As it does not have limits in the value of relative density. However, its major shortcoming is the computational cost. It is more expensive than other common approaches, especially when the problem contains a large number of variables [11,36]. Recently, a variational AH of beam-like square lattice structures has been discussed [34] and they explain and result when the microscale of the structure is in the finest scale, i.e., ϵ→0. Another computational approach is a matrix-based multiscale method introduced by Vigliotti et al. [24,37]. They performed a linear multiscale analysis and FEA (finite element analysis) on a stretching and bending-dominated lattice [37]. Furthermore, they have applied a method to develop a non-linear model for lattice materials [24].

Some homogenization approaches introduced here come from micro-polar theory [17,38,39,40] and solid-state physics [18,41]. The micro-polar theory introduces a microscopic rotation in addition to translational deformations. The micro-polar elastic constants of the stiffness matrix can be found through either analysis of the unit cell [17] or an energy approach [40]. From solid-state physics, the combination of Bloch’s theorem and the Cauchy–Born hypothesis has been applied to analyze mechanical behavior of planar lattices [18,41].

Recently, Machine Learning has been adopted to study lattice materials [36,42,43,44,45]. Koeppe et al. [36] have used a neural network on a set of simulation data to learn a parameterized mechanical model of a lattice structure with particular geometry. Mian et al. [42] obtained an elastic material model for lattice structure using both FEA (finite element analysis) and NN (Neural Network) approaches. These studies have produced results that are in good agreement with both experiment and simulation with a significant increase in computational time and prove that the data-driven method is an effective and efficient as well as reliable and accurate approach. In addition, Machine learning has been used to simulate anisotropic elastic-plastic behavior of cellular structure [45] and deep learning for topology optimization for lattice materials [44]. As machine learning and AI are developing in a rapid trend, data-driven methods are a rising prominent approach and worth looking into in the future of homogenization problems.

As has been briefly summarized above, many varieties of homogenization methods exist to analyze the behavior of lattice materials. All the methods mentioned came from various areas of discipline such as elasticity, solid-state physics, and even computer science. This implies the applications of lattice materials are substantial in many areas of science and engineering disciplines. The objective of this paper is to thoroughly review all the existing method that is relevant according to the author’s knowledge and interest. Its foundation, methodology, strength, and limitation will be discussed comprehensively here in a concise form. The final goal that this paper wants to achieve is for the reader to be able to carefully select their homogenization method based on its characteristic so that it could be applied optimally to each particular research.

## 3. Homogenization Methods

The fundamentals of the homogenization method are the properties of the heterogeneous material could be obtained from the analysis of a small portion of it [12]. The limited portion of the entire heterogeneous material is defined as Representative Volume Element (RVE). To obtain the effective properties, the *RVE* should include the main microstructural characteristic of the heterogonous material and expand to the global medium when uniform strain or stress is applied as boundary condition [12,46,47]. This avoids extensive full-scale simulations. Furthermore, it is noted that this method could be applied only if the homogeneities exist at least a couple of orders of magnitude below the characteristic length of the effective medium.

**Figure 5:**
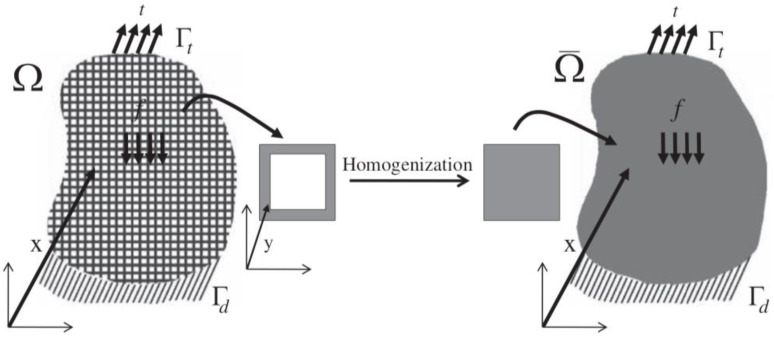
Homogenization concept of a cellular material [12].

The concept of homogenization of lattice materials is illustrated in Figure 5 where the *RVE* is applied to a square unit cell. A body Ω with a periodic lattice structure subjected to a traction t at the traction boundary $\Gamma_{t}$, a displacement d at the displacement boundary $\Gamma_{d}$, and a body force f is substituted by a homogenized body $\bar{\Omega }$. The mechanical properties of *RVE* should be determined in such a way that the macroscopic behavior of Ω and $\bar{\Omega }$ are equivalent [12]. Below is a detailed explanation of representative homogenization methods.

### 3.1. Beam Theory Approach

The Beam Theory approach is also known as the force-based approach [12]. It is employed to model cell-wall deformation just for a unit cell. Then it assumes that the field quantity obtained from the unit cell is uniform over the *RVE*. Over the years, analytical closed-form formula of the mechanical properties of lattice materials for different shapes and geometry has been derived [13,14,16]. Christensen [33] also gives a thorough survey on this approach.

**Figure 6:**
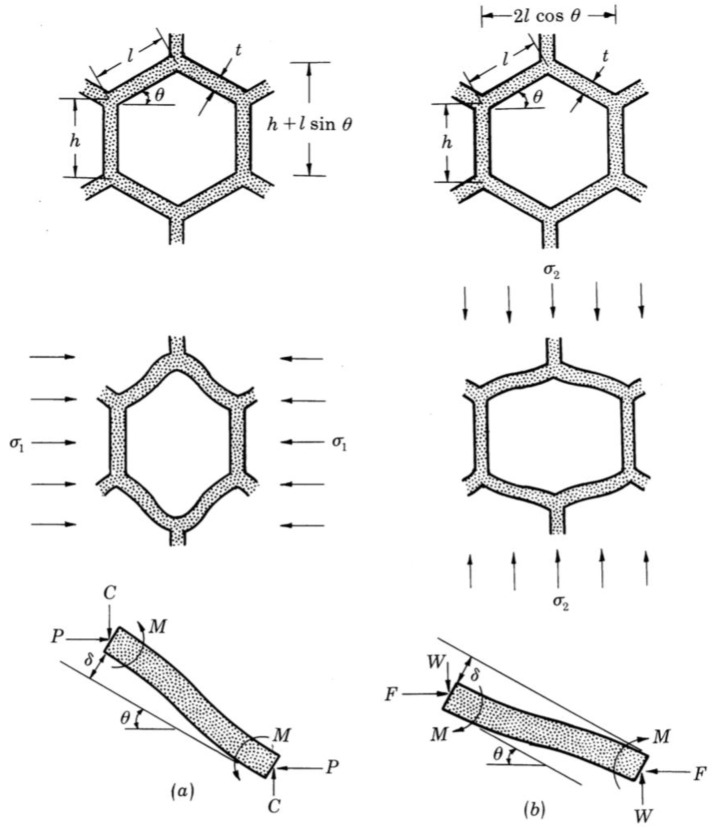
Beam theory analysis on honeycomb structure [13]. (**a**) and (**b**) represents structures under two different directional forces.

Gibson and Ashby are pioneers in the analysis of cellular materials, in particular of honeycomb shapes [13]. They analyzed the honeycomb shape by employing beam theory on a single unit cell as illustrated in Figure 6. They derived a closed-form solution of mechanical properties for honeycomb shape material and tested their formulation against experimental measurement under two different directional forces as depicted in Figure 6a,b. Masters and Evans [16] took it a step further where they included three mechanisms in their model, namely, flexure, stretching and hinging. They obtained a more general analytical expression for the mechanical properties. Wang and Mcdowell [14] investigated honeycomb structures with seven different cell types. They evaluated in-plane shear properties which had not been considered in most previous research.

Different treatments are expected for a different type of unit cell. For bending dominated lattice, the cell walls are treated as beams. Standard beam theory is employed here to calculate effective stiffness. In this case, the *linear-elastic* behavior is predominantly caused by the bending of the cell walls and edges, with minor contributions from shear and axial deformation. For stretching dominated lattice, the cell walls are treated as trusses/columns where the structure is capable of sustaining residual stresses that make equilibrium equations are insufficient to determine the state of internal forces on the cell walls. Additionally, compatibility equations should be used to find the effective elastic properties of the lattice. If the residual stress is assumed zero, then simple truss analysis suffices.

The main advantage of this approach is that the obtained mechanical properties are closed-form analytical formulas and they are useful to generate a chart. Assuming cell wall as a beam limits the applications of this method as follows: First, this formulation can be applied to only cases with low relative density ($\bar{\rho }<0.3$). Second, this approach cannot be used where geometrical nonlinearities are introduced or when the geometry of a unit cell has a complex topology as Euler’s beam formulation assume strains are small enough that large deformation does not occur.

### 3.2. Strain Energy Equivalence: Surface Average Approach and Volume Average Approach

Strain energy equivalence based method employs a direct application of the *RVE* concept. In this method, the performance of the macroscopic medium are determined solely by the mechanical behavior of its *RVE*. The averages of particular mechanical properties with respect to either the surface of the volume have to be equal in order to obtain the equivalence condition of effective medium and its *RVE* [48]. The constitutive equation for the effective medium and its corresponding *RVE* needs to be calculated in such a way that the condition for equivalence of both volume elements is satisfied.

The first approach for this method is the surface average approach. This approach uses the application of either stress or strain distributions to the surface of the *RVE* [12]. Hence, stress distribution in the *RVE* in assumed to be equivalent to a stress distribution in the volume element consisting of the effective medium if

(1) $$\int_{\Gamma_{RVE}^{i}}T_{i}d\Gamma_{RVE}=\int_{\Gamma_{RVE}^{i}}T_{i}^{*}d\Gamma_{RVE}$$

holds, where $T_{i}^{*}$ is the traction vector on the surface of the *RVE* and $\Gamma_{RVE}^{i}$ is a certain part of its boundary as the part which is orientated parallel to one of the coordinate planes. The second equivalence condition is between the strain tensor generated in the effective medium and its *RVE*, which can be expressed as

(2) $$\bar{\epsilon_{ij}}=\bar{\epsilon_{ij}^{*}}$$

Furthermore, for a volume element of general shape, the mesoscopic strain can be expressed by

(3) $$\bar{\epsilon }=\frac{1}{2}\frac{1}{V}\int_{\Gamma_{RVE}}\left(u_{i}n_{j}+u_{j}n_{i}\right)\;d\Gamma_{RVE}$$

wre V denotes the volume of the *RVE* and $n_{i}$ are the components of the normal vector on $\Gamma_{RVE}$. Equations (2) and (3) states that the surface integral of the quantity $\left(u_{i}n_{j}+u_{j}n_{i}\right)$ has to be equal for both volume elements.

Surface average approach has a certain limitation. For more complex geometry, such as those that are nonorthotropic, the surface average method gives errors in the prediction of the effective strain energy. This error is due to stress couples that are acting at the intersections of the cell walls and the surfaces of the *RVE*. In order to avoid this problem, a volume average approach can be used. The volume average approach is based on the assumption that the mechanical behavior of the microscopic scale in the *RVE* and the macroscopic medium can be considered equivalent if the *RVE* strain energy is equal to the effective medium. This can be expressed as

(4) $$\bar{w}=\frac{1}{V}\int_{\Omega_{RVE}}w\;d\Omega_{RVE}=\frac{1}{V}\int_{\Omega_{RVE}}w^{*}\;d\Omega_{RVE}=\bar{w^{*}}$$

where w denotes the strain energy density distribution and $\Omega_{RVE}$ is the area of the *RVE*. Thus, the strain equivalence condition can be written as

(5) $$\bar{\epsilon_{ij}}=\frac{1}{V}\int_{\Omega_{RVE}}\epsilon_{ij}\;d\Omega_{RVE}=\frac{1}{V}\int_{\Omega_{RVE}}\epsilon_{ij}^{*}\;d\Omega_{RVE}=\bar{\epsilon_{ij}^{*}}$$

Strain energy equivalence method has been commonly used in any kind of cellular structure such as sand which has a corrugated structure [48,49,50,51,52]. The advantage of this method is that it is directly based on the basic laws of continuum mechanics and the conservation of energy and of. Furthermore, there is no limitation in using this method in respect of geometries of the cellular structure and cell topology.

### 3.3. Micropolar Theory

Classical continuum theory is not suitable when discontinuities or high strain gradients are observed in the domain such as crack tips or notches. Micropolar theory, also known as Cosserat theory, is a generalization of classical continuum theory developed by E. and F. Cosserat [53] and Eringen [54]. The micropolar theory introduces a microscopic rotation in addition to translational deformations and its key assumption is both displacement and rotations of a point are independent kinematic properties. In lattice material, this means joint displacement and joint rotation contribute to the total joint displacement.

In the linear micropolar elasticity theory, the kinematic relations can be written as

(6) $$\epsilon_{ij}=u_{j,i\;}-e_{kij}\phi_{k}$$

(7) $$k_{ij}=\phi_{j,i\;}$$

where $u_{j,i}$ is the displacement gradient, $\epsilon_{ij}$ is the strain tensor, $\phi_{k}$ is the microrotation, $k_{ij}$ is the curvature strain tensor, and $\phi_{j,i}$ is the microrotation gradient. The generalized strain vector of a micropolar medium can be expressed as follows:

(8) $$\epsilon ={\left[\epsilon_{11}\;\epsilon_{22}\;\epsilon_{12}\;\epsilon_{21}\;k_{13}\;k_{23}\right]}^{T}={\left[u_{1,1}\;u_{2,2}\;u_{2,1}-\phi \;u_{1,2}+\phi \;\phi_{3,1}\;\phi_{3,2}\right]}^{T}$$

The generalized stress vector is given by

(9) $$\sigma ={\left[\sigma_{11}\;\sigma_{22}\;\sigma_{12}\;\sigma_{21}\;m_{13}\;m_{23}\right]}^{T}$$

where $m_{13}$ and $m_{23}$ are the couple stresses in the x and y planes. The 2D constitutive relations for anisotropic micropolar solids can be written as:

(10) $$\sigma =\bar{C}\epsilon$$

where $\bar{C}$ is the 6×6 matrix of the constitutive law coefficients for a micropolar medium.

In order to characterize a cellular material as a micropolar continuum, the coefficients of the constitutive equations, $\bar{C}$, must be obtained. The micropolar elastic constants of the stiffness matrix can be determined through either structural analysis of the unit cell [17] or an energy approach [40]. The analysis of the unit cell can be done using the beam theory approach to obtain the general deformation state of the *RVE*, which is a unit cell in this case. The effective stresses and strains over the *RVE* can be computed using constitutive equations. On the other hand, for the energy approach, the stresses of the cell can be obtained by obtaining the derivation of the strain energy density concerning the strain vector.

Micropolar theory combined with beam theory approach or energy approach has several limitations (1) It could only be applied to unit cells with a certain shape that contains a single joint at the center or the unit cell, and (2) the newly introduced micropolar variable acts as an additional degree of freedom. Hence, an additional step is required to solve the governing equations.

### 3.4. Solid-State Physics Approach: Bloch’s Theorem and Cauchy Born Hypothesis

Due to its similarity, the concept of solid-state physics can be adapted into solid mechanics to investigate the characteristics of lattice materials. The lattice, in solid-state physics, is defined as an infinitely periodic arrangement of points. When periods of the unit cell are perfectly stacked in two or three dimensions, the space is considered to be tessellated. The bases are the mathematical formulation for the physical quantities that are repeated in every cell translation [18]. In continuum mechanics, a lattice material can be described using the above definition.

Bloch wave analysis and the Cauchy–Born hypothesis, in particular, are methods for solid-state physics that can be adapted into solid mechanics to investigate the behavior of lattice materials [18,55]. Bloch’s theorem was originally developed to describe the transport of electron particles within the crystal structure of a solid [56]. Then the Bloch’s theorem can be applied to analyze the propagation of a wave function over to an infinite lattice structure. On the other hand, the Cauchy–Born hypothesis [41] analyzes a macroscopic mechanism that is induced by an applied strain [12] and states that the infinitesimal displacement field of a periodic lattice is made up of two parts, namely, the deformation obtained by a macroscopic strain field and the periodic displacement field of the unit cell. Bloch’s theorem is used to define the propagation of a wave function over the infinite lattice structure. The idea is that the nodal deformation function $d\left(p_{i},\omega \right)\in C^{2}$ is written as a wave function in the form of

(11) $$d\left(p,\omega \right)=d\left(j_{i}+\vec{R},\omega \right)=d\left(j_{l},\omega \right)e^{2\pi i\omega \vec{R}}\;\forall l\;\in \;\left\{1,2,\;\ldots ,J\right\}$$

where ω is the translational vector, p is the position vector for the joints, J is the number of independent nodes within the unit cell, $p_{i}=j_{i}+\vec{R}$ is the position vector of any node throughout the lattice and $\vec{R}$ is the Bravais cell vector of any unit cell through the entire lattice. For bar deformation functions, the generalized bar deformation vectors $e\left(q_{m},\omega \right)\in C^{2}$ can be written as a wave function of the form:

(12) $$e\left(q_{m},\omega \right)=e\left(b_{m}+\vec{R},\omega \right)=e\left(b_{m},\omega \right)e^{2\pi i\omega \vec{R}}\;\forall m\;\in \;\left\{1,2,\;\ldots ,B\right\}$$

where B is the number of independent bars within the unit cell and $q_{m}=b_{m}+\vec{R}$ is the position vector of any bar throughout the lattice. Periodic boundary conditions needs to be applied over the unit cell to simplify the forms of the kinematic and equilibrium matrices for both bars and joints [57,58].

Bloch’s theorem defines the deformation mechanism corresponding to periodic joint displacement fields. The Cauchy–Born hypothesis is needed to analyze the macroscopic strain field generated by periodic condition [59,60]. From the definition of the Cauchy–Born hypothesis [61], the infinitesimal displacement field of a periodic joint in a lattice structure can be expressed as:

(13) $$d\left(j_{l}+\vec{R},\bar{\epsilon }\;\right)=d\left(j,\bar{\epsilon }=0\right)+\bar{\epsilon }\cdot \vec{R}$$

where $d\left(j_{l}+\vec{R},\bar{\epsilon }\;\right)$ is the periodic displacement field of joint $j_{l}$. Assume that the periodic joints described by the position vectors $j_{l}$ and $j_{l}+\vec{R}$, are the two periodic joints i and j within a lattice structure, then Equation (13) can be written as:

(14) $$\left\lceil \begin{array}{c} u_{i}\\ v_{i} \end{array}\rceil =\lceil \begin{array}{c} u_{j}\\ v_{j} \end{array}\rceil +\left[\begin{array}{cc} \epsilon_{11} & \frac{1}{2}\;\epsilon_{12}\\ \;\frac{1}{2}\;\epsilon_{21} & \epsilon_{22} \end{array}\right]\lceil \begin{array}{c} x_{i}-x_{j}\\ y_{I}-y_{j} \end{array}\right\rceil$$

where u and v are the joint displacement components in the x and y directions, respectively, and joint i is the independent joint. Notice that the formulation above is written in terms of engineering strain. Equation (14) can be expressed as well as:

(15) $$\left\lceil \begin{array}{c} u_{i}\\ v_{i} \end{array}\rceil =\lceil \begin{array}{c} u_{j}\\ v_{j} \end{array}\rceil +\left[\begin{array}{ccc} \left(x_{i}-x_{j}\right) & 0 & \frac{1}{2}\left(y_{i}-y_{j}\right)\\ 0 & \left(y_{i}-y_{j}\right) & \frac{1}{2}\left(x_{i}-x_{j}\right) \end{array}\right]\lceil \begin{array}{c} \epsilon_{11}\\ \epsilon_{22}\\ \epsilon_{21} \end{array}\right\rceil$$

or in the shorter term:

(16) $$d_{i}=d_{j}+E\bar{\epsilon }$$

Equation (16) is the kinematic boundary condition of the Cauchy–Born hypothesis. Applying this boundary condition to the unit cell joint displacement vector, d, results in:

(17) $$d=T_{d}\tilde{d}+E\bar{\epsilon }\;$$

Substituting Equation (17) into the kinematic system of the unit cell results in:

(18) $$B\left\{T_{d}\tilde{d}+\tilde{E}\bar{\epsilon }\right\}=e$$

Equations (17) and (18) describe the application of the Cauchy–Born kinematic boundary condition to the continuum kinematic system of the lattice microstructure to express the relation between the microscopic displacements and a macroscopic strain field. The Cauchy–Born hypothesis cannot be applied to the kinematic compatibility relation of the unit cell without resorting to the Dummy node scheme [18]. This procedure, along with a more detailed derivation of this method, has been extensively discussed in previous literature [18,59,60,61] and will not be discussed here. This approach has been developed by assuming cell walls as beam elements. Hence, similar to the elasticity theory approach, these assumptions limit its application to low relative densities (ρ<0.3).

### 3.5. Asymptotic Homogenization Approach

Analytical solutions have shown some limitations in the applications of more general cases. Hence, one of the well-developed theories, with a sound mathematical foundation, that has been successfully used to predict mechanical properties in porous materials [35] is the Asymptotic Homogenization (AH) theory. This method has been validated with experimental results and proven to be a reliable and accurate method among them [62]. Arabnejad et al. performed extensive work on using AH to obtain mechanical properties of the lattice structure [11].

The pivot assumption of AH is that each physical quantity depends on two different scales: one on the macroscopic level x, and the other on the microscopic level, y=x/ϵ where ϵ is a ratio between *RVE* size and the size of the macroscopic medium means that stress/strain will vary faster by 1/ϵ. AH also assumes that field quantities change smoothly at the macroscopic level and have periodic condition at the microscale. Based on AH, each mechanical variable, such as the displacement field, u, can be expanded into power series concerning to ϵ:

(19) $$u^{\epsilon }=u_{0}\left(x,y\right)+\epsilon u_{1}\left(x,y\right)+\epsilon^{2}u_{2}\left(x,y\right)+\cdots$$

$u_{1}$ and $u_{2}$ are perturbations in the displacement field due to the microstructure and $u_{0}$ is the average value of the displacement field depend only on the macroscopic scale [35]. Take the derivative of the power series we get

(20) $$\frac{du}{dx}=\epsilon \left(u\right)=\frac{1}{2}{\left(\nabla u_{0}^{T}+\nabla u_{0}\right)}_{x}+\frac{1}{2}{\left(\nabla u_{1}^{T}+\nabla u_{1}\right)}_{y}+O\left(\epsilon \right)$$

(21) $$\epsilon \left(u\right)=\left\{\bar{\epsilon }\left(u\right)\right\}+\left\{\epsilon^{*}\left(u\right)\right\}\;$$

where $\bar{\epsilon }\left(u\right)$ is the macroscopic strain and $\epsilon^{*}\left(u\right)$ is the fluctuating strain at the microscopic level. Note that the terms of O(ϵ) and higher are neglected. Substitute the above equation into the weak form of equilibrium equation for a cellular body $\Omega^{\epsilon }$:

(22) $$\int_{\Omega^{\epsilon }}C_{ijkl}\left(\epsilon_{ij}^{0}\left(v\right)+\epsilon_{ij}^{1}\left(v\right)\right)\left({\bar{\epsilon }}_{kl}\left(u\right)+\epsilon_{kl}^{*}\left(u\right)\right)d\Omega^{\epsilon }=\int_{\Gamma }t_{i}v_{i}d\Gamma$$

where $C_{ijkl}$ is the effective stiffness tensor of the *RVE*, $\epsilon_{ij}^{0}\left(v\right)$ and $\epsilon_{ij}^{1}\left(v\right)$ are the macroscopic and microscopic strains, respectively, and t is the traction at the traction boundary $\Gamma_{t}$. The displacement v is selected to be contant on the macroscopic level and vary only on the microscopic level. Hence, this leads to:

(23) $$\int_{\Omega^{\epsilon }}C_{ijkl}\epsilon_{ij}^{1}\left(v\right)\left({\bar{\epsilon }}_{kl}\left(u\right)+\epsilon_{kl}^{*}\left(u\right)\right)d\Omega^{\epsilon }=0$$

Integrating over the *RVE* volume ($V_{RVE}$). Equation (23) may be rephrased as:

(24) $$\int_{V_{RVE}}C_{ijkl}\epsilon_{ij}^{1}\left(v\right)\epsilon_{kl}^{*}dV_{RVE}=-\int_{V_{RVE}}C_{ijkl}\epsilon_{ij}^{1}\left(v\right){\bar{\epsilon }}_{kl}dV_{RVE}\;$$

The equation above represents a local problem defined on the *RVE*. For a certain applied macroscopic strain, the material can be characterized if the fluctuating strain $\epsilon^{*}$ is known. The periodicity of the strain field is guaranted by applying periodic boundary conditions on the *RVE* edges; the displacements at opposite sides of the *RVE* are constrained to be equal [63]. The equation can be discretized and solved via FE analysis. For this objective, the equation needs to be simplified to obtain a relation between the microscopic displacement field and the force vector. This step will not be explained in this article. Instead, a simple example of solving an equation on one-dimensional domain will be illustrated below.

**Figure 7:**
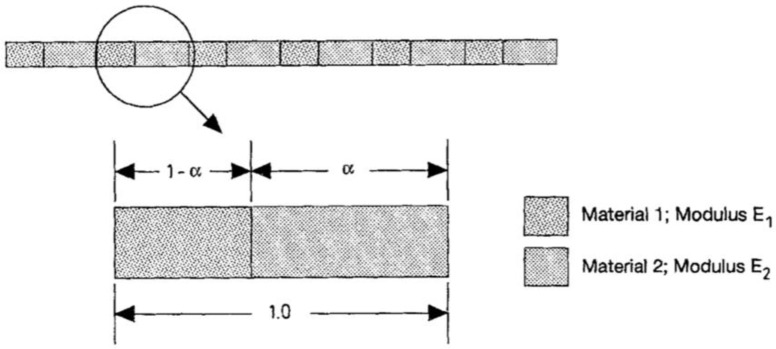
Composite bar used for the one-dimensional analysis [63].

Consider a composite bar consist of two materials that interchange periodically with Young’s moduli $E_{1}$ and $E_{2}$ which is described in Figure 7. Section 1 of the unit cell has material with a modulus $E_{1}$ and length 1−α. Section 2 of the unit cell has material with modulus E_2 and length α. The *RVE* on the microstructure of this case is chosen to be of unit length as is the area of the bar. Equation (24) in 1-D can be rewritten as:

(25) $$\int_{0}^{1}E\left(x,y\right)\epsilon^{*}\epsilon \left(v\right)dy=\int_{0}^{1}E\left(x,y\right)\epsilon \left(v\right)dy$$

where E(x,y) is Young’s modulus which varies at both the microscopic and macroscopic levels. First, set E(x,y)=E and rewrite Equation (25) as

(26) $$\int_{0}^{1}E\left(1-\epsilon^{*}\right)\epsilon \left(v\right)dy=0$$

Applying integration by parts

(27) $$\int_{0}^{1}v\frac{\partial }{\partial y}\;E\left(1-\epsilon^{*}\right)dy+E\left(1-\epsilon^{*}\right)v\;{|}_{y=0}^{y=1}=0$$

The strong form of Equation (27) is

(28) $$\frac{\partial }{\partial y}E\left(1-\epsilon^{*}\right)=0$$

Integrating gives the solution

(29) $$E\left(1-\epsilon^{*}\right)=c\left(x\right)$$

where c(x) is constant over the microstructure. To determine c(x), the equation is integrated over *y*

(30) $$\int_{0}^{1}\left(1-\epsilon^{*}\right)dy=\int_{0}^{1}\frac{c\left(x\right)}{E}\;dy\to 1-u^{*}\;{|}_{0}^{1}=c\left(x\right)\int_{0}^{1}\frac{1}{E}\;dy.\;$$

Since the displacements $u^{*}$ must be equal at the cell boundaries to ensure periodicity, thus

(31) $$c\left(x\right)=\frac{1}{\int_{0}^{1}\frac{1}{E}dy}$$

Hence, the effective stiffness can be expressed as

(32) $$\bar{E}=\int_{0}^{1}\left(1-\epsilon^{*}\right)dy=\int_{0}^{1}c\left(x\right)\;dy=c\left(x\right)=\frac{1}{\int_{0}^{1-\alpha }\frac{1}{E_{1}}dy+\int_{1-\alpha }^{1}\frac{1}{E_{2}}dy}$$

Evaluating the above integral gives us:

(33) $$\bar{E}=\frac{E_{1}E_{2}}{\left(1-\alpha \right)E_{2}+\alpha E_{1}}$$

Thus, for one-dimensional case, the effective stiffness obtained using AH method and the standard mechanics approach is equal.

The notable advantage of AH is that the stress distribution in microscale can be modeled accurately and thus give us a detailed analysis of the periodic materials. Furthermore, AH has neither limitation on the cell topology nor the range of the relative density which is a substantial gain of this method [11]. The major drawback of the AH method, however, is its computational cost. This can be a high problem if the problem involves complex topology and contains a significant number of variables.

### 3.6. Multi-Scale Homogenization Method for Lattice Materials

This approach is often called global-local analysis as it involves a two-scale process. This method is originally applied to heterogeneous material in order to create constitutive relationships from the analysis of the *RVE*. This method is developed based on the earlier work done by Eshelby [64] which investigated the mechanics of an ellipsoidal inclusion in an infinite matrix with homogeneous boundary conditions. The *RVE* features are somewhat similar to the ones that Elsheby has studied. It consists of a bounded area of the domain that contains the main microstructural properties of the material and behaves as an infinite medium if boundary conditions are imposed.

In general, this method utilizes a two-scale approach. One is the macroscopic FE model of the homogeneous continuum where boundary conditions are defined by the problem. The other is the microscopic level which numerically investigates the stress-strain relationship where boundary conditions are generated by the macroscopic scale. This approach allows the macroscopic stress to be determined as the gradient of the strain energy density involving the components of the macroscopic gradient. This approach results in a compact matrix formulation for the macroscopic stress as a function of the macroscopic displacement gradient.

**Figure 8:**
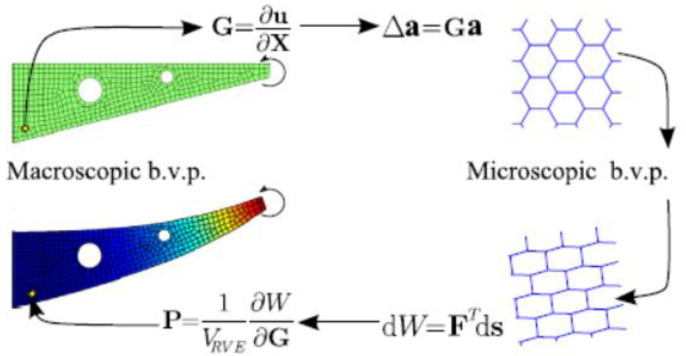
Multiscale scheme [24].

The method that is described here is the application multi-scale homogenization method to develop non-linear constitutive models for lattice materials [24]. This homogenization method is done using the principle of work which will be described shortly in this section. The details and derivation of this method can be found in the previous literature by Vigliotti et al. [24,37]. The main procedure for this method is described in Figure 8.

Let s be the vector of the nodal degree freedom of the *RVE*, the corresponding array of the nodal forces, F(s), can be obtained using FE analysis of the *RVE*. The distribution of the strain energy, due to macroscopic strain, can be obtained by employing the principle of the virtual work:

(34) $$dW=\int_{V_{RVE}}P_{ij}dG_{ij}dV=F^{T}ds$$

where $P_{ij}$ and $G_{ij}$ are the elements of the first Piola-Kirchoff (1PK) stress tensor and the macroscopic displacement, respectively; ds is the variation of the nodal displacements. Assuming that $P_{ij}$ and $G_{ij}$ are constant through out the *RVE*, the stress tensor can be obtained:

(35) $$P_{ij}=\frac{1}{V_{RVE}}\frac{\partial W}{\partial G_{ij}}=\frac{1}{V_{RVE}}F^{T}\frac{\partial s}{\partial G_{ij}}\;$$

Solving the equation above will introduce the boundary conditions for the microscopic model. Once the microscopic boundary value problem is solved, the components of P as the derivatives of the strain energy density of the lattice concerning G can be determined.

The main advantages of this method are that it accounts for geometrical material nonlinearity as have shown above and this approach has no restrictions in terms of relative density and unit cell shape. This model is capable to capture the local bucking of cell struts under multiple loading conditions and thus can predict the points where bifurcation occurs. However, unlike the AH approach, the choice of the *RVE*’s size might influence the equilibrium equation of the lattice especially in the presence of bifurcations [24]. Hence, a sensitivity analysis should be performed before choosing the size of the *RVE*.

### 3.7. Machine Learning Approach: Data-Driven Model

In recent years, there has been significant development of homogenization methods using machine learning algorithms [36,42,43,44,45]. Machine learning has been proven to be a dependable computational tool and employed in constitutive modeling [65,66,67,68]. As described in the previous section, while effective and precise, theoretical and numerical approaches each post major limitation. Theoretical approaches are limited for low relative density, small deformation, and simple geometry. Some of these limitations can be overcome using numerical approaches but these methods, such as FEA or AH, are computationally expensive. An alternative way is to use neural networks to do constitutive modeling based on either experiments or homogenization results as training data. In this section, we will discuss several strategies of implementation of this method that has been developed in recent years.

**Figure 9:**
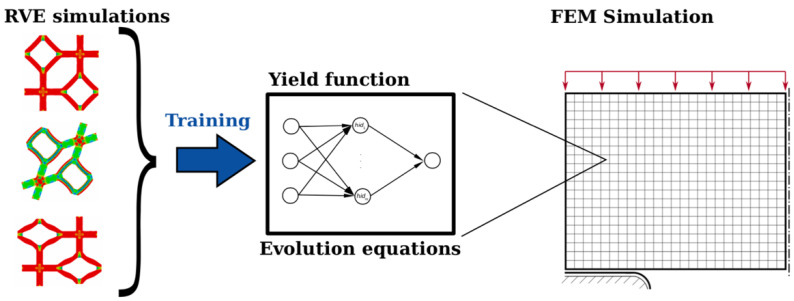
Graphical illustration of machine learning approach by Settgast et al. [45].

The fundamental initial phase of using machine learning algorithms, in this case, neural network approaches, is to generate training data. Either experimental data [65,68] or *RVE* simulations can be utilized for training process [45,66,67]. Settgast et al. [45] used the volume average method as their *RVE* simulation method and then used the results as the training data which is shown in Figure 9. The constitutive functions are obtained using neural networks instead of classic material modeling. FNET library is used to implement the neural networks [69]. Their study is limited to small deformation cases for simplicity but their approach can be straightforwardly extended to large deformation case. They can obtain an accurate result with much more efficiency than a direct numerical simulation (DNS) or FEM simulation.

**Figure 10:**
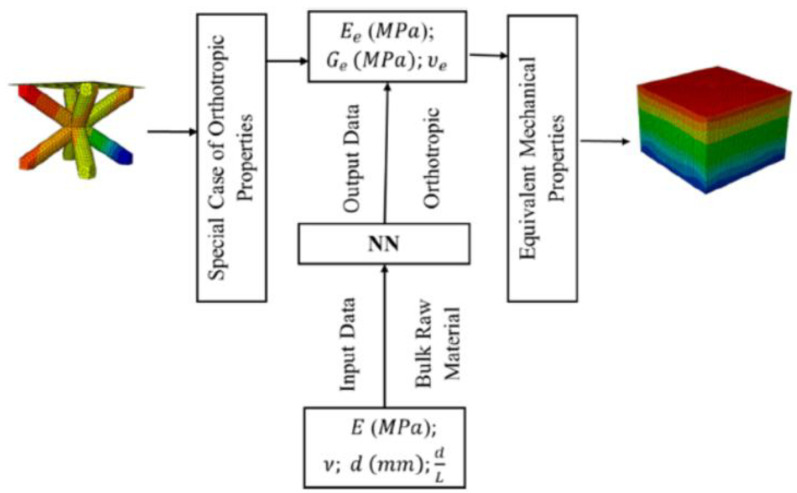
Integration of FEA model to NN model [42].

The other approach is to use finite element simulation (FEA) as the training data [42]. However, instead of full simulation of finite elements, only several models of lattice materials are simulated using FEA with a significant number of elements to compute the mechanical properties. Mechanical properties and design parameters data are used to train a NN to predict the equivalent properties for various cell sizes and materials with considerably less time than a full FE analysis. The result from this approach is compared with a full FEA simulation and experimental test. Their approach is briefly described in Figure 10. They concluded that the NN model of lattice materials is very accurate, swift, and efficient for use as compared to numerical FEA models. Furthermore, by using this approach a more complicated geometry of lattice can be investigated with significantly less computational time. It was shown that the computational time could be reduced from the order of hours to just order of minutes.

Another implementation strategy done by Koeppe et al. [36] is to combine experiments and finite element (FE) simulations to obtain training data. Firstly, lattice materials are created and tested under certain loading conditions. The experimental results will be validated against a parameterized FE model. Secondly, the developed FE model is utilized to predict the stresses considering different design variables. Finally, these deformations and design variables are used to train a NN to predict the stresses. This approach results in a significant increase in performance. The computation time for FE simulations is in order of five to ten hours (wall clock time) while the NN approach takes about 0.47 s. The obtained stresses by the neural network are in a good agreement with the FE results.

We try to point out each method’s main characteristic, its advantages, and limitation to give a concise comparison for the reader. The summary can be seen in Table 1.

## 4. Conclusions and Future Work

This paper has provided a concise review of several homogenization methods that can be applied to the analysis and design of lattice materials. These methods came from various areas of discipline such as elasticity, solid-state physics, and even computer science. Relative density, cell geometry, lattice category (structure or materials), and cell element assumptions have important roles in the behavior of lattice materials. Hence, it is critical to employ a proper model for the lattice regarding those parameters. A summary of each strength and weakness of each method has been shown in Table 1.

Out of all methods, due to its efficiency and accuracy, there has been a growing interest in the homogenization method using machine learning algorithms recently as it has proven to be a reliable computational tool and has been employed in constitutive modeling. Furthermore, it has been shown in the previous section how the machine learning approaches can overcome some major limitations that are posted by the classical homogenization technique.

Other than increasing efficiency, the recent and future works of homogenization are directed more towards the area of structure optimization. Homogenization coupled with optimization method has proven to increase both the efficiency of the optimization procedure and the overall performance of a lattice structure [70,71,72,73]. Stiffness [71], structural compliance [72], structural vibration [70] and energy absorption [73] have been proved to increase quite significantly using a homogenization method in a structural optimization procedure. It can be observed that most of these works use asymptotic homogenization as their method to be combined in the optimization procedure. As mentioned before, machine learning approach has a promising future in terms of its efficiency. Hence, it will be seen in the near future, integrated works of machine learning approach homogenization and optimization algorithm.

## Figures and Tables

**Table 1:** Summary of Homogenization Method.

Method	Underlying Theory	Highlights	Limitation
Beam Theory Approach [13,14,16,33]	Apply beam theory analysis on a single cell and assume uniform over the *RVE*	Close analytical formula. Relatively simple and does not need computational power.	Low relative density value (ρ<0.3). Simple topology Small strain and no large deformation.
Strain Energy Equivalence [48,49,50,51,52]	The averages of particular mechanical properties with respect to either the surface of the volume have to be equal in order to obtain the equivalence condition of effective medium and its *RVE*	Close analytical formula No restriction in terms of cell topology and its geometric symmetry	Small strain and no large deformation
Micropolar Theory [17,38,39,40]	Introduce a new variable, microscopic rotation, in addition to translational deformations and assume that both displacement and rotations of a point are independent kinematic quantities	Close analytical formula It does not need computational power	It needs to be combined with the beam theory approach or energy approach Only feasible for unit cells with a certain shape that contains a single joint at the center or the unit cell
Bloch’s Theorem and Cauchy–Born Hypothesis [18,55]	Bloch’s theorem is used to study the propagation of a wave function over an infinite lattice structure at a microscopic level. The Cauchy–Born hypothesis investigate macroscopic mechanisms induced by an applied strain.	Able to give a description of wave propagation over lattice structure Able to identify the collapse mechanism subject to macroscopic strain	Low relative density value (ρ<0.3)
Asymptotic Homogenization (AH) [11,35,62]	The main idea of AH is that each physical variables consist of two different scales: macroscopic and microscopic level.	No restriction on the unit cell geometry Works for all ranges of relative density Independent from *RVE* size	The computational cost is relatively expensive
Multi-Scale Homogenization Method [24,37,64]	This method utilizes a two-scale approach The macroscopic FE model of the component with certain boundary condition The microscopic level stress-strain relationship where boundary conditions are imposed by the macroscopic scale	No restriction on the unit cell geometry Works for all ranges of relative density Capable of capturing local bucking of cell walls under multiple loading conditions	The relatively expensive computational cost Depends on the *RVE* size. Hence, an additional convergence analysis needs to be done before using the method
Machine Learning Approach [36,42,43,44,45]	Use neural networks to do constitutive modeling based on either experiments or homogenization results as training data	Significantly low computational cost No limitation on cell topology and relative density	Needs to generate a huge amount of data to have an accurate result

## Data Availability

All data contained within the article.
